# Prevalence of isthmocele according to diagnostic hysteroscopy, transvaginal ultrasound, and magnetic resonance imaging in non-pregnant women: cross-sectional study

**DOI:** 10.1590/1806-9282.20260589

**Published:** 2026-08-10

**Authors:** João Paulo Leonardo-Pinto, Renata Telles Piva Belluomini, Luiz Gustavo Oliveira Brito, Jamal Mourad, Cristina Laguna Benetti-Pinto, Daniela Angerame Yela

**Affiliations:** 1 Universidade Estadual de Campinas, School of Medical Sciences, Department of Obstetrics and Gynecology – Campinas (SP), Brazil.; 2 Mayo Clinic Arizona, Division of Gynecological Surgery – Phoenix (AZ), United States.

**Keywords:** Ultrasonography, Hysteroscopy, Cesarean section, Magnetic resonance imaging

## Abstract

**OBJECTIVE:**

The aim of this study was to estimate the prevalence of isthmocele using transvaginal ultrasound, magnetic resonance imaging and diagnostic hysteroscopy.

**METHODS:**

A cross-sectional study conducted from 2020 to 2022 in tertiary academic hospital with ninety non-pregnant women with a history of cesarean section. Transvaginal ultrasound (n=90), magnetic resonance imaging (n=65) and office diagnostic hysteroscopy (n=81) were performed within a short interval for each patient.

**RESULTS:**

The women had a mean age of 34.8±6.3 years, mean number of 2.0±1.1 cesarean sections and mean duration of 40 months since their last cesarean. The prevalence of isthmocele with 95%CIs was 11.3% (5.5–19.7%) on transvaginal ultrasound, 66.1% (53.3–77.4%) on magnetic resonance imaging, and 43.2% (32.2–54.6%) on diagnostic hysteroscopy. When transvaginal ultrasound was considered reference, accuracy for diagnostic hysteroscopy was 57.89% (44.1%; 70.6%) and magnetic resonance imaging, 43.86% (30.9; 57.5%); when magnetic resonance imaging was reference, accuracy for diagnostic hysteroscopy was 54.39% (40.75; 67.43%). Agreement analysis (kappa) among the methods showed low scores between diagnostic hysteroscopy and magnetic resonance imaging regarding transvaginal ultrasound (0.126, p<0.001 and 0.103, p<0.001, respectively) and between diagnostic hysteroscopy and magnetic resonance imaging (0.206, p=0.014).

**CONCLUSION:**

The prevalence of isthmocele was lower in transvaginal ultrasound and higher in magnetic resonance imaging. Accuracy varied from 43 to 58% among the three methods and agreement between tests for diagnosing isthmocele was low.

## INTRODUCTION

Isthmocele is defined as a dehiscence at the site of a previous cesarean section scar, which acts as a reservoir collecting blood during the menstrual period, leading to abnormal uterine bleeding^1,2^. The prevalence of isthmocele varies from 19 to 84%, depending on the method used for diagnosis and when we individualize the diagnosis by hysteroscopy, we observe that data in the literature are scarce^3,4^. This variation is possibly due to differing definitions of isthmocele, the types of studies conducted, and the diagnostic methods employed^5,6^.

When assessed using transvaginal ultrasound (TVUS), isthmocele appears as an anechoic triangular area in the anterior region of the isthmus, easily detectable in non-pregnant women. Its shape and morphology can vary, presenting as a round, square, or wedge-shaped cavity. On diagnostic hysteroscopy (DxHyst), isthmocele appears as a cleft in the anterior uterine wall^7^. Magnetic resonance imaging (MRI) allows for clear delineation of the defect borders and measurement of myometrial thickness at the scar level. Despite several diagnostic techniques, there is no consensus on a gold standard for diagnosing isthmocele, although some studies propose standardizing classification using ultrasound^8,9^.

Despite several diagnostic techniques, there is no consensus on a gold standard for diagnosing isthmocele, although some studies propose standardizing classification.

The Diagnostic performance of 3D-TVUS does not appear to be significantly better than of 2D-TVUS^9^. However, both MRI and hysteroscopy, despite their higher costs, offer excellent accuracy^8,10,11^. Therefore, this study aims to estimate the prevalence of isthmocele using 2D-TVUS, MRI, and DxHyst, and to evaluate the accuracy and agreement between these.

## METHODS

We conducted a cross-sectional study at tertiary hospital, from November 2020 to December 2022. The study included 90 women with a history of cesarean section between 2015 and 2020, selected from a total of 452 women who had undergone cesarean sections. Of these, 277 were invited via telephone, email or during postpartum consultation. Women aged between 18 and 45 Women aged 18–45 years who had at least one cesarean section at least 6 months prior to study enrollment and within a maximum interval of 5 years were included. Pregnant women and those using hormonal or non-hormonal intrauterine devices were excluded. The study was approved by the Institutional Review Board of University of Campinas (CAAE 02238118900005404). The STROBE (Strengthening the Reporting of Observational Studies in Epidemiology) checklist for cross-sectional studies were followed.

Participants underwent two-dimensional TVUS and DxHyst by independent examiners on the same day to establish the diagnosis of isthmocele. MRI was performed the following day at an ultrasound clinic. Each exam was conducted by experienced physicians, each with at least 10 years of practice. The physicians were blinded to the results of the other examinations.

Data were collected on the following variables: age, body mass index (BMI), age at menarche, parity, presence and number of cesarean sections, time elapsed since the last cesarean section, and the indication for cesarean section (elective or emergency).

Technical details about the instruments have been previously described in a published study with the same cohort of women to assess risk. Two-dimensional TVUS was performed using Toshiba Xario or Voluson E8 devices to evaluate the uterus and adnexa. The isthmocele was assessed for its width (W), depth (D), length (L) and residual myometrial thickness (RMT). MRI was performed using the Achieva 1.5 Tesla equipment (Philips, Eindhoven, Netherlands) with a six-receptor coil dedicated to high-resolution pelvic examinations. The exams were conducted with patients in a supine position, with moderate bladder repletion with no vaginal gel. The diagnosis of other uterine pathologies and the signal intensity of the contents of the endometrial cavity and isthmocele contents were described. Parameters regarding the length (L), depth (D), width (W), and RMT of the isthmocele were measured. Diagnostic hysteroscopy was performed on an outpatient basis without anesthesia, following the technique described by Bettocchi and Selvaggi in 1997^12^, using saline solution as the distension medium. The isthmocele was evaluated for the presence of tent-shaped retraction on the anterior wall of the myometrium at the isthmus and the presence or absence of edge hypertrophy in this area.

### Statistical analysis

Sample size calculation was described in a previous publication analyzing factors associated with isthmocele^13^. Since the primary outcome for this study differs from the previous one, we used a convenience sample. In the previous publication, a composite variable derived from all three exams was used to calculate the global prevalence of isthmocele. However, since this study aims to identify the prevalence of isthmocele from each of the three exams individually, the composite variable was not used here. Descriptive analysis was performed to evaluate the frequency, means and standard deviations of the variables. Prevalence was displaying as proportion plus 95%CIs. Given the absence of a gold standard diagnostic test for isthmocele, 2D-TVUS was used as the to calculate the sensitivity, specificity, positive predictive value, negative predictive value and accuracy plus 95%CIs of the tests. To evaluate the agreement between the methods, the kappa coefficient kappa coefficient was calculated, and the McNemar test was used to compare the prevalence of isthmocele between the methods in related samples. Statistical analysis was performed using SAS version 9.4 (Cary, NC, USA) with a significance level of 5%.

## RESULTS

Table 1 shows the sociodemographic characteristics of the patients. The mean age was 34.8±6.3 years and the mean number of cesarean sections was 2.0±1.1, with 55.6% of women having two or more cesarean sections. The time elapsed from the last birth to inclusion was 40.1±14.7 months.

**Table 1:** Demographic characteristics of enrolled women (n=90).

	Mean±SD	n (%)
Age (years)	34.8±6.3	
Race		
White		65 (72.2)
Black		7 (7.8)
Others		18 (20)
BMI (kg/m^2^)	30.7±6.9	
Menarche (years)	12.6±1.6	
Parity	3.0±1.5	
Cesarean section	2.0±1.1	
Number of c-section		
1		40 (44.4)
2		23 (25.6)
3		17 (18.9)
≥4		10 (11.1)
Time elapsed last c-section (months)	40.1±14.7	
Moment for indication of cesarean section		
Elective		50 (55.6)
Urgency		40 (44.4)

SD: standard deviation; BMI: body mass index.

The prevalence of isthmocele varied according to the diagnostic method: 11.3% (5.5–19.7%) with TVUS, 66.1% (53.3–77.4%) with MRI and 43.2% (32.2–54.6%) with hysteroscopy. The mean uterine volume was 111.3±57.7 cm^3^ for TVUS and 98.3±44.7 cm^3^ for MRI (p=0.542). The cesarean section scar was identified in 100% of MRI exams and 71% of TVUS exams. Hysteroscopy found that 85% of anteverted uteri were anteverted and 10 participants had their exams abbreviated due to pain or cervical stenosis. RMT was greater than 3 mm in 42 (97.6%) subjects with isthmocele. Regarding isthmocele measurements, TVUS showed a larger width than MRI; all other measures were similar (Table 2).

**Table 2:** Assessment of transvaginal ultrasound, magnetic resonance imaging, and diagnostic hysteroscopy for isthmocele.

	TVUS (n=90)		MRI (n=65)		Hysteroscopy (n=81)	
	Mean±SD	n (%)	Mean±SD	n (%)	Mean±SD	n (%)
Uterus						
Volume (cm^3^)	111.3±57.7		98.3±44.7		-	
Longitudinal (cm)	84.3±15.0		82.7±10.4		-	
Anteroposterior (cm)	45.5±12.0		47.6±9.6		-	
Transversal (cm)	50.9±10.5		45.6±9.3		-	
Caesarean section scar						
Full scar		68 (71.2)		65 (100)		-
Isthmocele		10 (11.3)		43 (66.1)		35 (43.2)
Isthmocele						
Width (mm)	11.0±3.8		5.2±2.0			
Amplitude (mm)	7.5±5.8		4.0±1.5			
Depth (mm)	7.9±6.9		3.9±1.3			
RMT (mm)			4.9±1.5			
RMT ≥3 mm				42 (97.6)		

SD: standard deviation; RMT: residual myometrial thickness; TVUS: transvaginal ultrasound; MRI: magnetic resonance imaging.

Table 3 compares the three diagnostic methods for isthmocele. MRI and DxHyst had the highest sensitivity values, and DxHyst had the highest accuracy rates. However, the agreement rate for diagnosing isthmocele among the methods was low. When comparing MRI results to TVUS (used as the reference in this study), there was a significant difference in the prevalence of isthmocenle on MRI (p<0.001) with an accuracy of 43.86% (30.98%; 57.57%). When comparing hysteroscopy to TVUS, a higher prevalence was also found with hysteroscopy (p<0.001) and an accuracy of 57.89% (44.13%; 70.60%). When comparing the two most accurate methods, hysteroscopy and MRI, there was still low agreement between them (p=0.014). MRI had an accuracy of 54.39% (40.75%; 67.43%) when compared to hysteroscopy (Table 3).

**Table 3:** Assessment of diagnostic methods for isthmocele and agreement between them.

	Sensitivity (95%CI)	Specificity (95%CI)	PPV (95%CI)	NPV (95%CI)	Accuracy (95%CI)	Kappa (95%CI)	p***
MRI (TVUS)*	88.89 (50.67; 99.42)	35.42 (22.55; 50.60)	20.51 (9.87; 36.94)	94.44 (70.2; 99.71)	43.86 (30.98; 57.57)	0.103 (-0.018; 0.225)	<0.001
Hysteroscopy (TVUS)*	66.67 (30.92; 90.96)	56.25 (41.28; 70.23)	22.22 (9.38; 42.73)	90.00 (72.32; 97.38)	57.89 (44.13; 70.60)	0.126 (-0.071; 0.324)	<0.001
Hysteroscopy (MRI)**	88.24 (62.25; 97.94)	40.00 (25.28; 56.61)	38.46 (23.81; 55.35)	88.89 (63.93; 98.05)	54.39 (40.75; 67.43)	0.206 (0.032; 0.380)	0.014

TVUS: transvaginal ultrasound; PPV: positive predictive value; NPV: negative predictive value; MRI: magnetic resonance imaging; CI: confidence interval.

*TVUS was adopted as a reference;

**MRI was adopted as a reference;

***McNemar test.

## DISCUSSION

In our study, the prevalence of isthmocele varied according to the diagnostic method with MRI and hysteroscopy presenting the highest proportions (66 and 43%). MRI had an accuracy of 43.8% and the hysteroscopy of 57.89% considering TVUS as a reference. There was low agreement between diagnostic methods.

We evaluated the accuracy of three methods—TVUS, MRI, and DxHyst., compared in pairs for diagnosing isthmocele. This study is one of the few that assess all three methods for this disorder. However, our results did not show high accuracy rates compared to some other studies in the literature.

Most of the recommendations suggest that TVUS should be the starting point for diagnosing isthmocele, as acquiring the simple sagittal plane and performing contrast sonohysterography are straightforward and require only medium-level diagnostic skills^9,14^. TVUS can be complemented by contrast-enhanced sonohysterography, using intrauterine instillation of saline solution or gel. Second-generation contrast agents that have been tested during contrast-enhanced US have shown superior accuracy with conventional 2D TVUS, according to a recent systematic review^15^. In the current literature, as well as in our study, prevalence rates vary according to the diagnostic method, ranging from 24 to 70% with TVUS and from 56 to 84% with sonohysterography^14,16^. Antila-Långsjö et al, in a study carried out with 371 women undergoing TVUS and sonohysterography, observed that the isolated use of TVUS failed to diagnose about half of the isthmoceles in this population^10^. Similarly, in our study, compared with MRI, only a quarter of isthmoceles were identified on TVUS.

Variation in the prevalence rates of isthmocele by TVUS may depend on the examiner’s experience, the complexity of the lesions, the type of suture, and the time elapsed since the cesarean section and diagnosis. A recent study found a 42.3% prevalence of isthmocele six weeks after c-section and an association between locking continuous sutures and the presence of more niches^16,17^.

TVUS isthmocele dimensions were similar to MRI measurements, except for the isthmocele ´width. A recent study analyzed whether these dimensions could predict the ability to confirm isthmocele and found that defect length and width significantly contributed to the diagnosis^18^.

MRI is an expensive exam and usually has higher sensitivity and accuracy rates than TVUS for diagnosing isthmocele. Our accuracy rates were lower than usual, but the prevalence rates were similar to previous studies^19^. Generally, MRI accuracy rates are higher than TVUS^19,20^. Despite its higher cost, MRI, like hysteroscopy depends on more advanced technology but is more accurate than TVUS^7,8,19^. Gupta et al. did not find a statistically significant difference between MRI and TVUS for diagnosing isthmocele^19^. We used a 1.5 T MRI to detect isthmocele. Although we did not compare it to other MRI technologies, such as 3.0 T, a recent study using 3.0 T MRI found an accuracy of 92.86% compared to 69.64% for TVUS^20^. Moreover, MRI derived parameters may be used to differentiate an abnormal post-cesarean uterine scar from a normal one, similar to our study’s findings^21^.

Most of the studies have addressed hysteroscopy as both a diagnostic and treatment method for isthmocele. It is a well-established method for evaluating the uterine cavity^22,23^. An advantage of DxHyst is its ability to exclude other endometrial pathologies that could cause similar symptoms. However, a disadvantage is that it cannot measure the thickness of the residual myometrium over the niche^24,25^. Despite this, its accuracy rate is similar to MRI.

The study has several limitations. The small sample size, resulting from the recruitment phase during the COVID-19 pandemic, might have increased the odds of a type 2 error. Additionally, this was a single-center study, which may limit external generalizability. There is also potential response bias as we do not have the profile of non-responders to the study invitation.

Therefore, we propose the need for new further, prospective, cohort studies to define the most appropriate diagnostic method for identifying isthmocele. TVUS should be performed by two different operators to calculate inter-variability influence, with similar time intervals from the last cesarean section, and from different centers. Larger samples would allow for a more comprehensive study of this subject, using statistical tools to correct for confounding bias, such as propensity score analysis. Comparisons with MRI and hysteroscopy should also be included to develop a more specific algorithm for diagnosing this disorder. As the literature suggests, TVUS has good diagnostic test parameters and should be the primary choice for investigating isthmocele. Additionally, studies focusing on the prevention of isthmocele should be planned, given the rising cesarean section rates worldwide.

## Data Availability

The datasets generated and/or analyzed during the current study are available from the corresponding author upon reasonable request.
